# Nanoparticle-Based Systems for Delivery of Protein Therapeutics to the Spinal Cord

**DOI:** 10.3389/fnins.2018.00484

**Published:** 2018-07-19

**Authors:** Juan C. Infante

**Affiliations:** Department of Molecular and Cellular Biology, Harvard College, Harvard University, Cambridge, MA, United States

**Keywords:** protein therapeutics, drug delivery systems, nanoparticles, spinal cord injuries, FGF1, Liraglutide

## Abstract

Recent studies have demonstrated that delivery of protein therapeutics to the spinal cord may promote functional axon regeneration, providing a pathway for recovery of certain motor skills. The timeframe for delivery of protein therapeutics, however, must be modulated to prevent bulk release of the therapeutics and minimize the frequency of implantations. This perspective examines both affinity-based and nanoparticle-based strategies for delivery of neurotrophic factors (NFs) to the spinal cord in an effective, safe, and tunable manner.

## Introduction

Spinal cord injuries (SCIs) often have debilitating effects on patients and can severely impact quality of life. Each year there are about 20,000 new injuries and currently there may be up to 350,000 SCI patients in the United States (Spinal Cord Injury (SCI) Facts and Figures at a Glance, 2016). Finding a treatment for these injuries is therefore of utmost importance and axon regeneration may prove to be one of the most promising alternatives.

Several groups have shown that inducing axon regeneration is in fact possible. A 2010 study found that following peripheral nerve injury, mice that were locally treated with insulin-like growth factor 1 (IGF-1) displayed significant gains in axon number and diameter (Apel et al., 2010). Other studies have also found that IGF-1 can enhance axon growth in cultured corticospinal motor neurons (Ozdinler and Macklis, 2006; He, 2010). In fact, it is thought that IGF-1 plays a crucial role in CNS development since double-knockout IGF-1 mice display problems in the creation of functional nerve connections (Ozdinler and Macklis, 2006). IGF-1 and other protein therapeutics are therefore promising options for the treatment of SCIs.

However, in order for axon regeneration to be clinically meaningful in the context of paralysis, it must be accompanied by at least some functional recovery of motor skills. Most studies up to date have been unable to induce significant elongation in severed or injured CNS motor axons (Axon Regeneration in the Central Nervous System, 2016). Since these axons are usually only able grow a few millimeters, functional recovery is difficult to achieve. A 2015 study found that co-expression of IGF1 and osteopontin (OPN) could promote axon regeneration in retinal ganglion cells (Duan et al., 2015; He and Jin, 2016). It has also been shown that overexpression of fibroblast growth factor 1 (FGF-1) in the spinal cord improves functional recovery after SCI, as measured by the Basso-Beattie-Bresnahan locomotion scale (Li et al., 2018). Liraglutide, a glucagon-like peptide 1 analog that is already an approved treatment for type 2 diabetes, has similarly been implicated in functional recovery (Chen et al., 2017). FGF-2, brain-derived neurotrophic factor (BDNF), and neurotrophin-3 (NT-3) have also been shown to enhance motor neuron outgrowth following injury (Boyce and Mendell, 2014; Santos et al., 2016). In fact, some research suggests that synergistic administration of NFs may be most beneficial (Logan and Ahmed, 2006). Thus, the question becomes whether such protein therapeutics can be reliably delivered to the spinal cord. Studies have successfully delivered protein therapeutics, and seen associated functional improvements, through implantation of adeno-associated virus (AAV) vectors into the spinal cord (Li et al., 2018). These findings are very promising in terms of their potential applications to patients in the short-term future. However, constant AAV implantations as a form of human treatment seem to be impractical. This perspective thus examines both affinity-based and nanoparticle-based strategies for delivery of neurotrophic factors (NFs) to the spinal cord in an effective, safe, and tunable manner.

## Affinity-Based Systems

**FIGURE 1:**
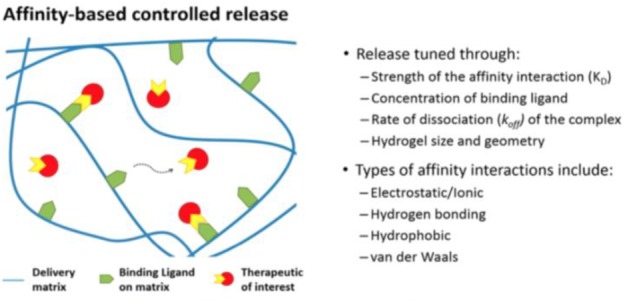
Schematic of principles behind Affinity-Based Release Systems (Vulic and Shoichet, 2014) – Published under ACS Author Choice License, which permits copying and redistribution of the figure for non-commercial purposes.

There are two different approaches for delivering FGF-1, Liraglutide, and other NFs in general. The first one involves an affinity-based delivery system (**Figure 1**). This system consists of a polymeric matrix that has a ligand for our therapeutic of interest immobilized on the matrix. The rate of release of the therapeutic can therefore be controlled by modulating the dissociation kinetics of our protein therapeutic and its matrix-immobilized ligand (Vulic and Shoichet, 2014). Several heparin-based delivery systems (in which heparin is the ligand) have been studied to date. FGF-2 release from heparin-gelatin-PEGDA gels occurred over a 42-day timeframe, but this matrix is not biocompatible (Vulic and Shoichet, 2014). Heparin-based biocompatible matrices have achieved delivery of FGF-2 over comparable timeframes (Vulic and Shoichet, 2014). Similarly, heparin-based release of NT-3 and BDNF has been sustained for 14 and 7 days, respectively (Vulic and Shoichet, 2014). However, affinity-based systems exhibit certain limitations: mainly that protein therapeutics may be inadvertently immobilized or that ligand-protein binding may sterically hinder release of the therapeutic (Vulic and Shoichet, 2014). Similarly, the fact that practical ligands, such as heparin, can bind multiple therapeutics, or that some therapeutics such as IGF-1 must first bind binding proteins before binding to heparin, introduces too many layers of control into these systems and adds unnecessary complexity to the modulation of dissociation kinetics. Therefore, it is useful to explore the benefits that nanoparticle-based delivery systems provide.

## Nanoparticle-Based Systems

The second approach involves a nanoparticle delivery system which essentially modulates release of the protein therapeutics by degradation of the particle matrix. The proteins are encapsulated in the nanoparticles and, after an initial period of rapid release, are slowly released as the particle matrix progressively degrades (Vulic and Shoichet, 2014). However, issues surrounding this approach include the use of organic solvents, such as chloroform, that are not only toxic but may also denature the therapeutic of interest (Vulic and Shoichet, 2014). In addition, usual nanoparticle preparation protocols involve extensive use of sonication which may lead to protein aggregation and thus damaging of a protein’s bioactivity (Stathopulos et al., 2004; Vulic and Shoichet, 2014). However, nanoparticle-based systems may be able to sustain protein release for more than two weeks, despite the rapid release period in the first 24 h (Swed et al., 2014). This release-profile appears promising for translational studies since the goal is to reduce the frequency of injections and implantations as much as possible. The following section therefore proposes, in detail, future experimental directions for designing non-toxic nanoparticle delivery systems that may modulate the slow release of NFs without compromising their bioactivity.

### Experimental Strategy

The first challenge when designing nanoparticle systems involves ensuring that the therapeutics of interest are successfully encapsulated. Initial investigations could prepare basic water-in-oil micelles to gauge the efficacy of protein encapsulation through standard procedures. A fluorescent protein, GFP for instance, could be encapsulated inside a micelle using chloroform, even though this organic solvent could never be used *in vivo* due to its toxicity. Since GFP is soluble in water, one would expect to observe fluorescence in the aqueous layer only if the protein was successfully encapsulated in the micelle. Successful encapsulation could suggest that hydrophilic proteins may be stably enclosed in hydrogels and other similar systems. We were able to conduct this experiment and will discuss the results in the following sections.

Further investigations could involve the preparation of poly-lactic-co-glycolic acid (PLGA), a biodegradable material, nanoparticles using a non-separation method with non-toxic, and non-denaturing, organic solvents (Swed et al., 2014). Glycofurol (GF) and isorbide dimethyl eher (DMI), presumably non-toxic and non-denaturing solvents, may be independently used to determine whether one of them leads to more effective encapsulation of hydrophilic fluorescent proteins of similar weights to the NF of interest (outline in Appendix) (Tran et al., 2012; Swed et al., 2014). It is crucial to use proteins that have similar weights since this can have a significant effect on the diffusion rate of the therapeutic (Swed et al., 2014). The size differences between FGF-1 and Liraglutide, for instance, could pose a potential problem when attempting to co-deliver these proteins as therapeutics, since the MW of FGF-1 is about 17 kDa while that of Liraglutide is about 4 kDa. It would not be as ideal to have different release rates for FGF-1 and Liraglutide, although this could perhaps be attenuated by using some of the principles in affinity-based systems (perhaps by using heparin or some other ligands) to modulate release rates. The main feature of this preparation method is that sonication is not needed and the risk for protein aggregation can therefore be minimized (Tran et al., 2012).

After encapsulation of the therapeutic of interest, it is necessary to verify its structural integrity to ensure that no major denaturation took place. Protein samples released within the first 24 h can be collected in a phosphate buffer (would not degrade the protein) and compared to wild-type protein through circular dichroism (CD) spectroscopy (Swed et al., 2014). The release profile of the protein therapeutic can then be estimated by measuring fluorescence intensity of protein samples in the phosphate buffer at different time-points (each day until no more apparent changes in intensity can be detected).

### Micelle Results

No fluorescence was detected in the aqueous layer. This result can be explained by multiple factors, ranging from the use of chloroform as the organic solvent to the need for sonication in the protocol. As previously mentioned, certain organic solvents may cause proteins to denature. It is possible that chloroform caused GFP to denature, which could possibly prevent the protein from fluorescing. This does not necessarily mean that encapsulation was unsuccessful, although it would not be very useful to deliver a denatured protein that has lost its bioactivity. Alternatively, it is also possible that the lack of fluorescence was due to a failure in encapsulation. Another possibility is that aggregation of GFP led to quenching of the fluorescence signal, since, as mentioned before, sonication may lead to protein aggregation (Stathopulos et al., 2004). Although our experiments with these basic micelles proved to be unsuccessful, it would be interesting to determine whether the use of non-toxic, non-denaturing procedures would yield positive results.

### PLGA Expected Results

#### CD Spectroscopy

**FIGURE 2:**
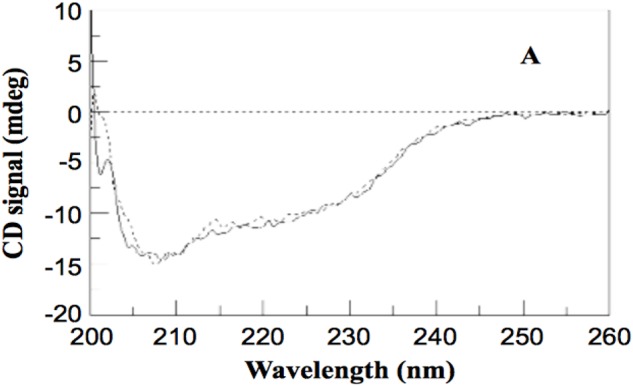
CD Spectrum showing intact structural integrity of an encapsulated protein relative to WT (Swed et al., 2014) – Published under Creative Commons Attribution License, which permits unrestricted use, distribution, and reproduction of figure in any medium.

Since the protein is expected to be successfully encapsulated in the PLGA nanoparticle without being denatured, the CD spectra of the released protein (found in the phosphate buffer) and the wild-type protein should be identical (**Figure 2**) (Swed et al., 2014). This would indicate that the structure of the protein did not change during encapsulation and that the protein likely retained its bioactivity.

#### Fluorescence Analysis

When analyzing the protein samples in the phosphate buffer, fluorescence intensity should gradually increase each day until no more changes can be detected. At this point, it is reasonable to assume that the majority, if not all the protein, has already been released from the nanoparticle. The rate of change of the fluorescence intensity can then be used to estimate the rate of release of the protein therapeutic.

## Discussion

Recent literature suggests that nanoparticle delivery systems may be quite effective for patient applications. For our purpose, the blood brain barrier (BBB) poses an extra difficulty. The nanoparticles must not only be non-toxic and able to modulate the slow release of the protein-therapeutic, but must also be small enough to cross the BBB. PLGA nanoparticles may be the solution to all three problems. First, PLGA is particularly useful because it is a biodegradable material, which addresses toxicity concerns. Second, previous studies have been able to prepare PLGA nanoparticles for drug delivery in the 200-nm range, which is required for the particles to cross the BBB (Tamilselvan et al., 2014). Finally, some PLGA-based delivery systems have sustained release of therapeutics for up to 30 days, providing a reasonable timeframe for translation to patients; a timeframe which may further improve once regulation is fine-tuned (Vulic and Shoichet, 2014). Nanoparticle delivery methods prove to be a promising alternative for delivering protein therapeutics that may promote functional axon regeneration. If the discussed nanoparticle systems can be effectively used in a mouse model, clinical trials may be coming soon.

## Author Contributions

The author confirms being the sole contributor of this work and approved it for publication.

## Conflict of Interest Statement

The author declares that the research was conducted in the absence of any commercial or financial relationships that could be construed as a potential conflict of interest.

## Appendix

### – PLGA Protocol

**Adapted from (Tran et al., 2012; Swed et al., 2014).**

**Non-Toxic PLGA Protein Encapsulation**

**Materials**

• Any hydrophilic florescent protein similar in size to IGF1
• Glycofurol (GF)
• Isosorbide dimethyl ether (DMI)
• Uncapped 75/25 PLGA
• Lutrol F-68
• Ethanol
• Glycine buffer
• NaCl and Tris–HCl solutions
• Phosphate-buffered saline

**Procedure 1 (2 weeks)**

• Preparation of Protein Precipitates (Suspension of Protein Precipitates)
  – A total of 10 μg fluorescent protein dissolved in 2M NaCl solution containing 15% w/v Lutrol F68.
  – Mixture of 120 μl GF and 75 μl DMI were added to 5 μl of Tris–HCL 0.05M? first solution was then added to this.
• Preparation of Polymer Solution
  – Prepare 12% w/v solution of PLGA in GF.
  – Leave mixture under stirring for 48 h and then leave to stand for 7 days at RT? You should observe a macroscopic evolution of polymer solution.
  – Before any further use, filter solution through a 0.2-μl pore size filter.
• Preparation of Protein-Loading Particles
  – First, add 100 μl of protein precipitates into 200 μl of polymer solution to obtain a suspension of protein precipitates in polymer solution.
  – The 50–150 μl of ethanol were added into the mixture, right before 0.9–1.8 ml of 1% F-68 solution was added into the mixture to start the phase separation (Solution X).
  – A total of 15 ml of 1% Lutrol solution in glycin buffer 1.25 mM pH (9–11) introduced (Solution Y).
  – After 15 min, 15–25 ml of Solution Y added into the suspension and this suspension was left to stand for 12–24 h? At the end, the pH should be around 7.
  – Successively centrifuge at 1000 × *g* for 30 min and then 2000 × *g* for 30 min? Eliminate the supernatant? Final volume should be about 1 ml.
  – Freeze-dry the suspension

**Procedure 2 (2 weeks)**

• Preparation of Protein Precipitates
  – Fluorescent protein dissolved in 0.16M NaCl solution to obtain concentration of 20 mg/ml. A total of 25 μl of this solution was then added to 975 μl of glycofurol (GF).
• All other steps the same

**Analyzing Structural Integrity**

• Protein samples released within 24 h into phosphate-buffered saline containing 0.1% w/v Lutrol F68 concentrated to 0.06 mg/ml.
• Compare circular dichroism (CD) spectrum of released and wild-type protein spectra should be the same.

**Estimating Rate of Release**

• Track fluorescence intensity each day. Fluorescence intensity should gradually increase each day until no more changes can be detected.
